# The metabolism of 1,25(OH)_2_D_3_ in clinical and experimental kidney disease

**DOI:** 10.1038/s41598-022-15033-9

**Published:** 2022-06-28

**Authors:** Mandy E. Turner, Tyler S. Rowsell, Christine A. White, Martin Kaufmann, Patrick A. Norman, Kathryn Neville, Martin Petkovich, Glenville Jones, Michael A. Adams, Rachel M. Holden

**Affiliations:** 1 Department of Biomedical and Molecular Sciences, Queen’s University, 3048C Etherington Hall, Kingston, ON K7L 3V6 Canada; 2 Department of Medicine, Queen’s University, Kingston, ON K7L 3V6 Canada; 3 Kingston General Health Research Institute, Kingston Health Sciences Centre, Kingston, ON K7L 3V6 Canada; 4 Department of Public Health Sciences, Queen’s University, Kington, ON K7L 3V6 Canada

**Keywords:** Endocrinology, Nephrology

## Abstract

Chronic kidney disease (CKD) results in calcitriol deficiency and altered vitamin D metabolism. The objective of this study was to assess the 24-hydroxylation-mediated metabolism of 25(OH)D_3_ and 1,25(OH)_2_D_3_ in a cross-sectional analysis of participants with a range of kidney function assessed by precise measured GFR (mGFR) (N = 143) and in rats with the induction and progression of experimental kidney disease. Vitamin D metabolites were assessed with LC–MS/MS. Circulating measures of 24-hydroxylation of 25(OH)D_3_ (24,25(OH)_2_D_3_:25(OH)D_3_) precisely decreased according to mGFR in humans and progressively in rats with developing CKD. In contrast, the 1,24,25(OH)3D3: 1,25(OH)_2_D_3_ vitamin D metabolite ratio increased in humans as the mGFR decreased and in rats with the induction and progression of CKD. Human participants taking cholecalciferol had higher circulating 1,24,25(OH)_3_D_3_, despite no increase of 1,25(OH)_2_D_3_. This first report of circulating 1,24,25(OH)_3_D_3_ in the setting of CKD provides novel insight into the uniquely altered vitamin D metabolism in this setting. A better understanding of the uniquely dysfunctional catabolic vitamin D profile in CKD may guide more effective treatment strategies. The potential that 24-hydroxylated products have biological activity of is an important area of future research.

## Introduction

The 2017 KDIGO guidelines for the management of chronic kidney disease-mineral bone disorder (CKD-MBD) suggest that patients with rising PTH values be investigated for modifiable factors, including vitamin D deficiency^1^. The clinical assessment of vitamin D deficiency is a measurement of 25-hydroxyvitamin D (25(OH)D), which captures dietary intake of precursor molecules and endogenous ultraviolet light-mediated generation^2^. 25(OH)D_3_ is converted to 1,25(OH)_2_D_3_, by the cytochrome P450 enzyme, CYP27B1, which is up-regulated by PTH and down-regulated by fibroblast growth factor 23 (FGF-23) and 1,25(OH)_2_D_3_^2^. In contrast to the production of 25(OH)D, the catabolism of 25(OH)D is tightly regulated and an essential step is 24-hydroxylation, catalyzed by CYP24A1^3^. CYP24A1, found in tissues where the VDR is expressed, controls local levels of 1,25(OH)_2_D_3_. Catabolism of 1,25(OH)_2_D_3_ to 1,24,25(OH)_3_D_3_ is also performed by CYP24A1, but whether this pathway is altered in CKD has not yet been studied.

Using liquid chromatography tandem mass spectroscopy (LC–MS/MS), the vitamin D metabolome assessed in this study consists of the main circulating form, 25(OH)D_3_, the hormonally active product of CYP27B1 (1,25(OH)_2_D_3_), and the initial hydroxylation products of CYP24A1 (24,25(OH)_2_D_3_ and 1,24,25(OH)_3_D_3_). An estimate of the catabolic equilibrium of 25(OH)D_3_ and 1,25(OH)_2_D_3_ was assessed by calculating the 25- and 1,25-vitamin D metabolite ratios (VMRs): 24,25(OH)_2_D_3_:25(OH)D_3_ and 1,24,25(OH)_3_D_3_:1,25(OH)_2_D_3_.

The objective of this study was to evaluate the vitamin D metabolome across a range of kidney function, in (1) a cross-sectional study of participants with measured GFR (mGFR) and (2) longitudinally in rats with induction and progression of CKD.

## Methods

### Vitamin D metabolite measurement

Serum vitamin D metabolites were measured by LC–MS/MS (Acquity LC/Xevo, TQ-S system; Waters Corp.) as previously described^4–6^. For measurement of 1,25(OH)_2_D_3_ and 1,24,25(OH)_3_D_3_, 150 µL of serum was equilibrated with 200 pg/mL d6-1,25(OH)_2_D_3_ and 12.5 pg/mL d6-1,24,25(OH)_3_D_3_ internal standard. The sample was incubated with 100µL of anti-1,25(OH)_2_D_3_ antibody slurry (Immundiagnostik) for 2 h at RT with orbital shaking at 1200 RPM. The slurry was isolated by vacuum filtration and rinsed with 4 × 400 µL of water, and vitamin D metabolites were eluted with 2 × 200 µL ethanol. The eluate was dried and derivatized with DMEQ-TAD as previously described^4^. The sample was re-dissolved in 50 µL 50/50 (% by vol) methanol/water and 35 µL was injected into the LC–MS/MS system as previously described^7^. MRM transitions used for analysis of 1,25(OH)_2_D_3_ and 1,24,25(OH)_3_D_3_ were m/z 762- > 468 + 762- > 484, and m/z 778- > 468 + 778- > 484. Quantification was based on a 6-point calibrator generated in-house containing 5–300 pg/mL 1,25(OH)_2_D_3_ and 1–25 pg/mL 1,24,25(OH)_3_D_3_. The LoQ for 1,24,25(OH)_3_D_3_ was 5 pg/mL with a coefficient of variation of 10–15%. Both humans and animal models were measured using this method.

### Human study

We measured the vitamin D metabolome in participants of 2 cohort studies (Supplementary Fig. 1). All subjects were aged > 18 and had been recruited from Kingston Health Sciences Centre and the general population of Kingston, Ontario Canada. All participants gave informed consent according to the Declaration of Helsinki and both study protocols were approved by Queen’s University and Associated Teaching Hospitals Research Ethics Boards. Demographic data as well as medications and supplement use were obtained by chart review and participant interview at the time of sample collection. The GFR Measurement Study included a convenience sample of 98 community dwelling participants ≥ 18 years of age. We excluded kidney transplant recipients (n = 14), those without biological sample (n = 9), participants taking calcitriol (n = 6) and those with substantial circulating vitamin D_2_ (n = 3), as defined by > 15% of circulating total 25(OH)D, indicating consumption of plant-based vitamin D supplements. The metabolic products of vitamin D_2_ are not captured by the assay used in this study. The second study was the Healthy Aging Study and included 78 healthy participants between the ages of 40 and 80 with targeted recruitment of 10 males and 10 females within each decade of life. We excluded 1 participant from the Healthy Aging Study with substantial circulating vitamin D_2_. Participants from two studies were pooled for a total convenience sample size of 143 participants.

GFR was measured either by urinary inulin clearance (N = 51) or iohexol plasma clearance (N = 84), as previously described^8,9^. Inulin-based GFR was measured via urinary clearance averaging 3 1-h periods. Iohexol-based GFR determination measured plasma clearance hourly 2–4 h following iohexol administration, with a Brochner-Mortensen correction to account for the initial clearance phase^10^. GFR was corrected for body surface area (BSA). Nine participants had technical errors with iohexol and inulin measurement and creatinine-based CKD-EPI eGFR of less than 45 mL/min/1.73 m^2^ but were included in grouped assessments as creatinine-based GFR approximations are more accurate at lower levels of GFR^11^. Serum creatinine, phosphate and calcium were measured at Kingston Health Sciences Centre Core Laboratory (Roche Plus Modular). Intact-PTH was measured using an ELISA (Immunotopics, Inc., Quidel). Urine microalbumin (LoQ 5.1 mg/L) was measured on Abbott ARCHITECT c systems c16000 with CV < 5%. Intact-FGF-23 was measured in duplicate via ELISA, in fifty-six samples (Kainos Laboratories ELISA kit), as per instructions.

### Rat study

Animal procedures were in accordance with the Canadian Council on Animal Care and approved by Queen’s University Animal Care Committee. Male Sprague–Dawley rats (n = 18, Hilltop Lab Animals Inc, PA, USA) were 15 weeks of age at the time of induction of CKD. Prior to intervention, animals were acclimatized for a week whilst housed individually and maintained on a 12-h light/dark cycle. All animals were provided with a diet containing 0.5% phosphate, 1% calcium, 0.05% magnesium, 0.2 mg/kg vitamin K, 1 IU cholecalciferol and 6% protein (Harlen Tekland WI, USA TD 150555). CKD was induced in 10 animals with the addition of 0.25% adenine to the diet weeks whilst the parallel group of non-CKD controls (n = 6) remained on the same non-adenine diet. If animals did not eat all administered food or reached 10% weight loss, they were supplemented with a high calorie supplement (Boost, Clear H2O, USA). Animals were blood sampled from the saphenous vein.

Serum creatinine and serum and urine calcium and phosphate were evaluated spectrophotometrically (SynergyHT Microplate Reader; BioTek Instruments, Winooski, VT). Creatinine was evaluated using the Jaffe method (QuantiChrom™ Creatinine Assay Kit, Bioassay Systems). Calcium was measured using the o-cresolphthalein complexone method at 540 nm, as previously described (Sigma-Aldrich, CAN)^2^. Free phosphate was measured using the malachite green (Sigma-Aldrich, CAN) method as described by Heresztyn and Nicholson^3^ at 650 nm. Plasma levels of intact PTH and intact FGF-23 were measured using ELISAs (Immunotopics Inc., USA). The measurement of the serum vitamin D metabolites was identical to the procedures outlined above.

### Statistical analysis

Data were analyzed using SAS software (V9.4), SAS/STAT software (V14.2) and GraphPad Prism (V8.4.2). The Shapiro–Wilk test was used to assess normality and several variables were log-transformed as a result. Participants were stratified into 15 mL/min/1.73 m^2^ mGFR intervals. Groups were compared using a Mann–Whitney U-test or ANOVA. Pairwise comparisons were completed between mGFR groups using Fisher’s LSD test in variables for which the global test was significant. Spearman’s correlation coefficient was used to evaluate the association between vitamin D metabolites and demographic and laboratory measures, with subsequent partial associations consecutively controlling for age and mGFR. The threshold for significance for all p-values was 0.05. Vitamin D metabolites in rats were compared using repeated measures one-way ANOVA with post hoc test for differences between adjacent time points.

## Results

### Participant characteristics

The 143 human participants had a range in mGFR from 8.4 to 128 mL/min/1.73 m^2^.and were predominantly white (97.2%), with mean age of 59.7 ± 12.4 years, body mass index (BMI) of 28.4 ± 5.9 kg/m^2^, and a prevalence of diabetes of 16.1%. Table 1 stratifies the participants by mGFR categories of 15 mL/min/1.73 m^2^. Participants with lower mGFR were older, had slightly higher BMI but similar BSA, and were more likely to be diabetic. Consistent with CKD progression, there was an increase in albuminuria, phosphate, PTH, and FGF-23 as mGFR declined.

**Table 1:** Demographics of participants stratified by measured GFR.

mGFR (mL/min/1.73 m^2^)	GFR categories							P-value
	ALL (n = 143)	> 90 (n = 12)	60–89 (n = 70)	45–59 (n = 13)	30–44 (n = 12)	15–29 (n = 26)	< 15 (n = 10)	
**Demographics**								
Age	59.71 ± 12.37 (19.00–88.00)	45.83 ± 12.36(25.00–64.00)	57.86 ± 11.74(19.00–76.00)***	63.00 ± 9.68 (46.00–76.00)***^,†^	66.92 ± 5.62 (56.00–78.00)***^,††^	65.73 ± 11.32 (37.00–88.00)***	60.80 ± 13.36 (39.00–78.00)**	** < 0.001**
Race (N, % white)	139 (97.2%)	12 (100.0%)	69 (98.6%)	13 (100.0%)	12 (100.0%)	24 (92.3%)	9 (90.0%)	0.332
Sex (N, % female)	67 (46.9%)	4 (33.3%)	38 (54.3%)	5 (38.5%)	5 (41.7%)	11 (42.3%)	4 (40.0%)	0.648
Diabetic (N, % yes)	23 (16.1%)	0 (0.0%)	2 (2.9%)	1 (7.7%)	4 (33.3%)^††^	12 (46.2%)**^,†††,‡^	4 (40.0%)*^,†††^	** < 0.001**
**CKD biomarkers**								
uACR (n = 105)	2.03 [0.93, 16.3]	2.07 [0.79, 5.10]	1.04 [0.71, 2.26]	1.40 [1.00, 8.25]	8.05 [1.20, 16.20]	12.60 [2.03, 120.8]^†††^	160.3 [81.10, 340.2]***^,†††,‡‡,&&,$^	** < 0.001**
Serum Ca (mM) (n = 133)	2.29 ± 0.11	2.32 ± 0.06	2.30 ± 0.10	2.33 ± 0.14	2.32 ± 0.13	2.27 ± 0.1	2.20 ± 0.16	0.066
Serum PO_4_ (mM) (n = 132)	1.19 ± 0.25	1.11 ± 0.11	1.13 ± 0.15	0.99 ± 0.16^*†*^	1.11 ± 0.29	1.34 ± 0.22**^,†††,‡‡‡,&&^	1.52 ± 0.46***^,†††,‡‡‡,&&&,$^	** < 0.001**
PTH (pmol/mL) (n = 140)	6.35 [4.70, 10.03]	6.75 [4.40, 7.99]	5.50 [4.30, 6.50]	7.40 [5.30, 9.80]	6.0 [3.92, 7.34]	10.46 [7.66, 15.74]*^,†††,&&^	23.31 [16.70, 29.80]***^,†††,‡‡‡,&&&,$^	** < 0.001**
FGF-23 (pmol/L) (n = 56)	10.75 [7.11, 18.24)	3.79 [3.09, 4.48]	4.23 [2.35, 8.11]	6.42 [3.67, 7.22]	10.83 [8.03, 15.06]^†^	12.42 [8.71, 24.00]*^,†††,‡‡^	36.26 [23.37, 46.85]**^,†††,‡‡,&^	** < 0.001**

Data represented by mean ± SD for normally distributed measures or median [IQR] for non-normally distributed. P-value indicates significance of one-way ANOVA or non-parametric equivalent. Differences between groups evaluated using Fisher LSD test if global test has p < 0.05.

*uACR* urinary albumin-to-creatinine ratio, *PTH* parathyroid hormone, *FGF-23* fibroblast growth factor 23.

*p < 0.05, **p < 0.01, ***p < 0.001 indicates significance compared to > 90, ^†^60–89. ^‡^45–59, ^&^30–44, ^$^ < 15.

Significant values are given in bold.

### Vitamin D metabolites and ratios delineated by kidney function

**Figure 1:**
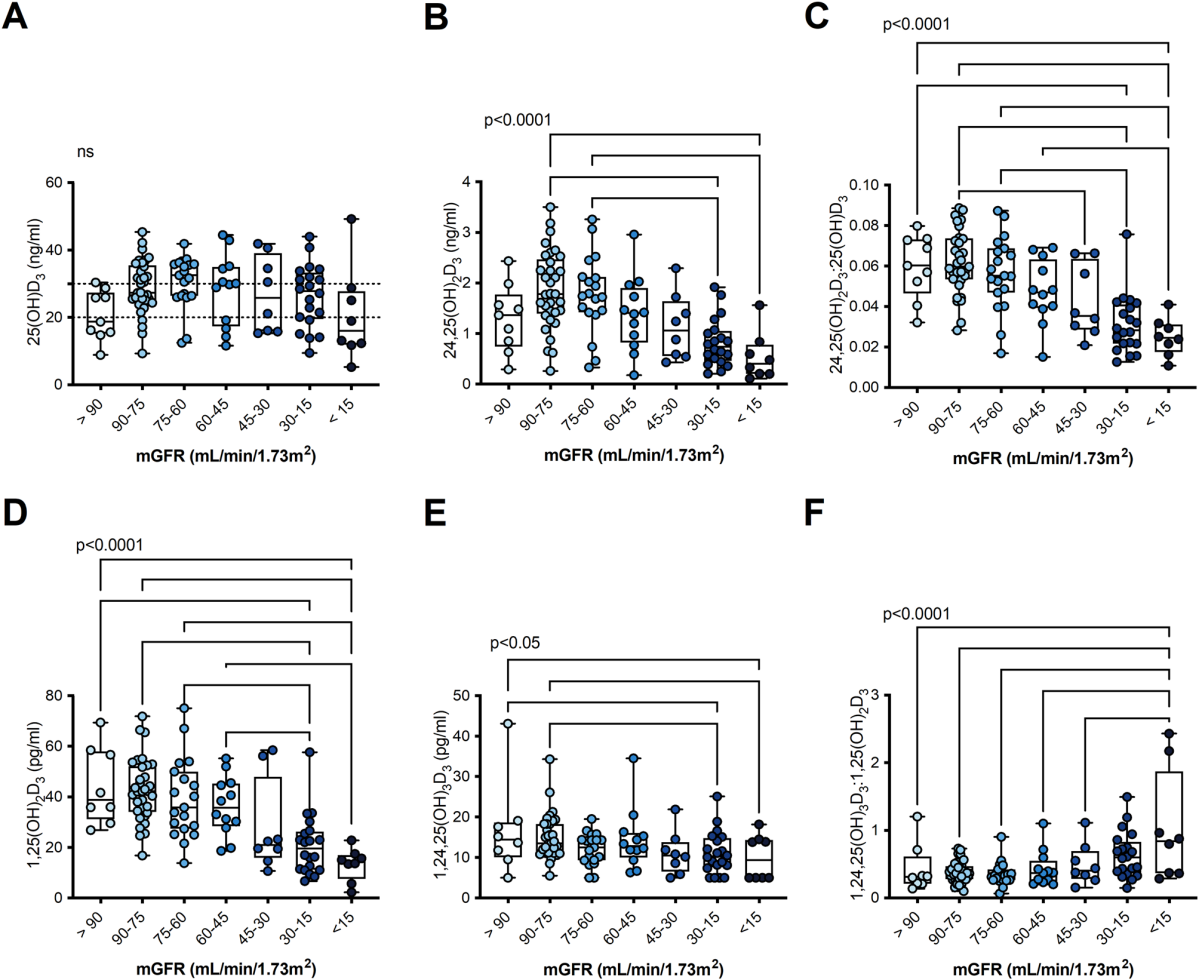
Vitamin D metabolites and 24-hydroxylation ratios in participants delineated by GFR measured by iohexol/inulin clearance. (**A**) 25(OH)D_3_, (**B**) 24,25(OH)_2_D_3_, (**C**) 25-VMR 24,25(OH)_2_D_3_:25(OH)D_3_, (**D**) 1,25(OH)_2_D_3_, (**E**) 1,24,25(OH)_3_D_3_, (**F**) 1,25-VMR 1,24,25(OH)_3_D_3_:1,25(OH)_2_D_3_. Dotted lines on (**A**) indicate cut offs for insufficiency and deficiency. One-way ANOVA with post hoc test for linear trend between mGFR categories, if mGFR categories were a significant source of variation, pairwise comparisons were performed with Fishers LSD test for (**A**–**F**). *p < 0.05, **p < 0.01, ***p < 0.001, ****p < 0.0001. Box plot indicates medial, IQR and range. Participants reporting cholecalcierfol use excluded from graphs and analysis (> 90 N = 109; 90–75 N = 33; 75–60 N = 19; 60–45 N = 12; 45–30 N = 8; 30–15 N = 20; < 15 N = 9).

The level of 25(OH)D_3_ was similar across the range of kidney function (Fig. 1A). As kidney function declined, 24,25(OH)D2D3 (Fig. 1B) and the 25-VMR (24,25(OH)_2_D_3_:25(OH)D_3_) decreased (Fig. 1C). 1,25(OH)_2_D_3_ and 1,24,25(OH)_3_D_3_ both decreased as kidney function declined (Fig. 1D,E) however, the 1,25-VMR (1,24,25(OH)_3_D_3_:1,25(OH)_2_D_3_) showed an increasing trend (Fig. 1F). Both with and without adjustment for mGFR and age, a higher level of PTH was associated with a lower 24,25(OH)_2_D_3_:25(OH)D_3_ ratio. In contrast, FGF-23 was not associated with the 25-VMR but higher level of FGF-23 was associated with an increase in the 1,24,25(OH)_3_D_3_:1,25(OH)_2_D_3_ ratio. (Table 2). The vitamin D metabolites and their ratios were highly associated with each other, even after adjustment for mGFR and age (Supplementary Table 1).

**Table 2:**
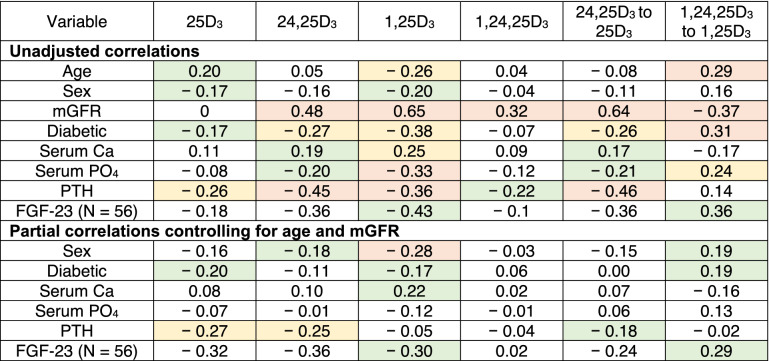
Spearman correlations of vitamin D metabolites with demographics and serum measures in participants unadjusted and partially adjusted controlling for age and mGFR.

Spearman r value indicated. Colour delineates p-value: Red < 0.001, Yellow < 0.01, Green < 0.05.

**Figure 2:**
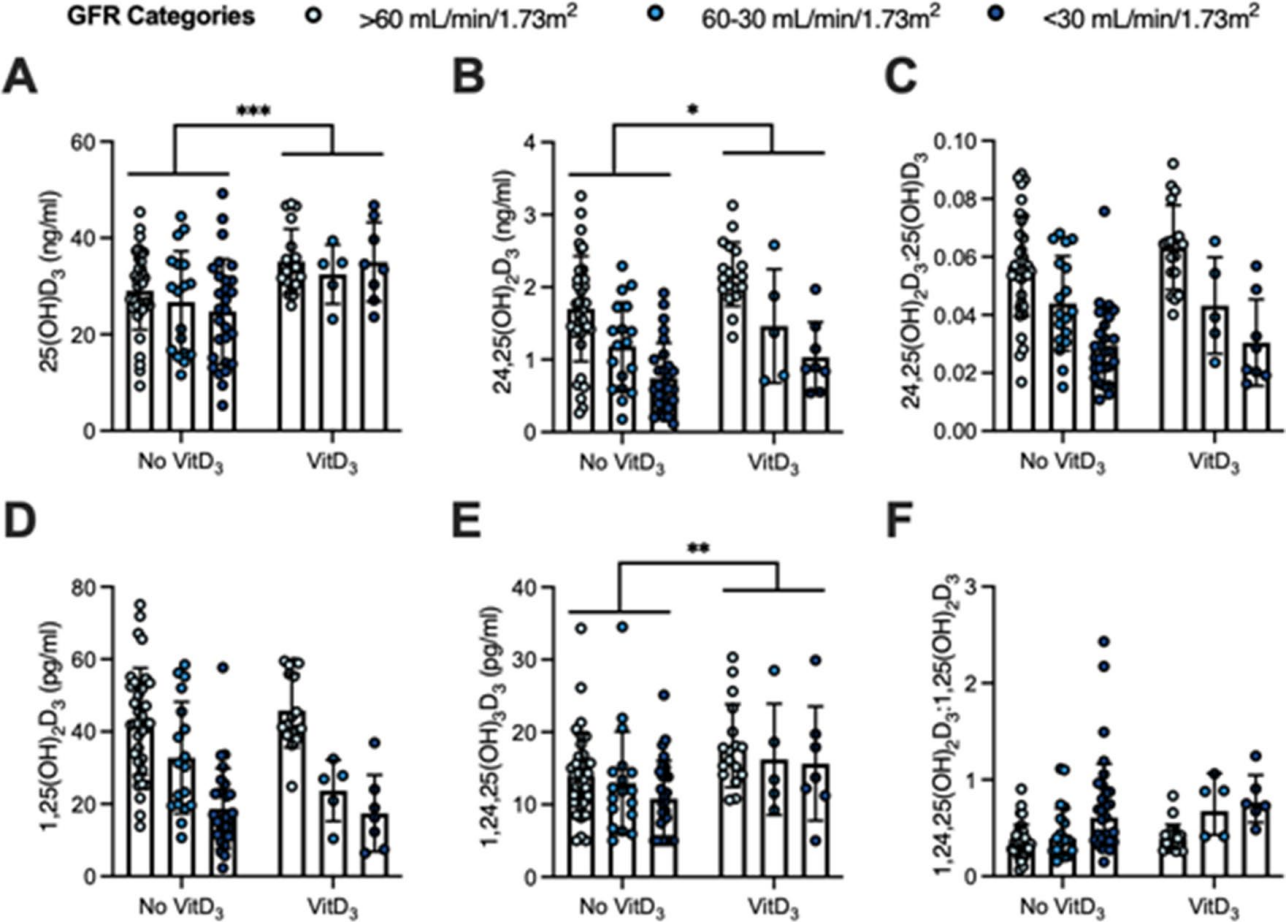
Vitamin D metabolites separated by cholecalciferol use delineated by measured GFR. (**A**) 25(OH)D_3_, (**B**) 24,25(OH)_2_D_3_, (**C**) 25-VMR: 24,25(OH)_2_D_3_:25(OH)D_3_, (**D**) 1,25(OH)_2_D_3_, (**E**) 1,24,25(OH)_3_D_3_, (**F**) 1,25-VMR: 1,24,25(OH)_3_D_3_:1,25(OH)_2_D_3_, Two-way ANOVA. Mean SD. P-value indicates whether cholecalciferol use was a significant source of variation. *p < 0.05, **p < 0.01, ***p < 0.001.

Thirty-four (24%) participants self-reported taking cholecalciferol at the time of blood sampling (Fig. 2). The median dose was 1000 IU/day, ranging from 400 to 10,000 IU/day. We found higher levels of 1,24,25(OH)_3_D_3_ in participants taking cholecalciferol with no elevation in 1,25(OH)_2_D_3_. The level of 25(OH)D_3_ was higher overall in participants taking cholecalciferol regardless of kidney function, yet the 25-VMR ratio did not change.

### Animal study

**Figure 3:**
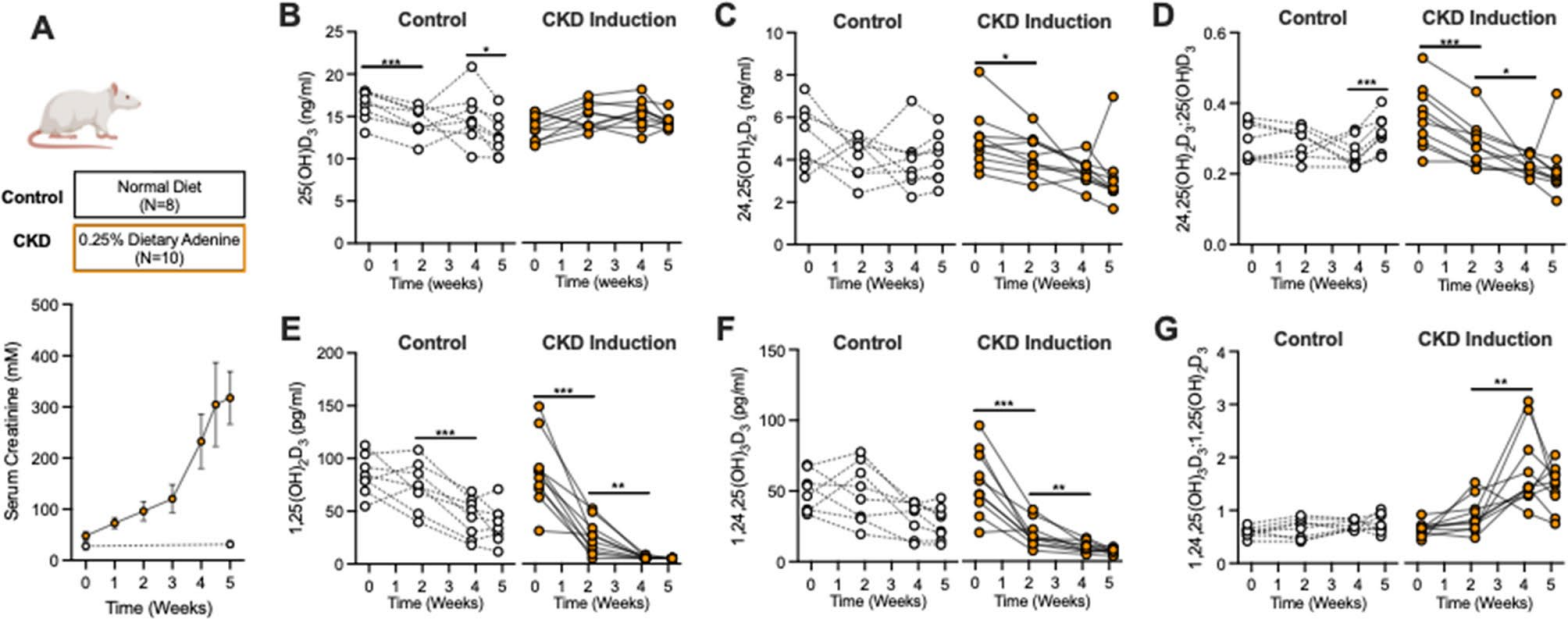
Longitudinal vitamin D measurements from a rat model of CKD mirrors human 1,24,25(OH)_3_D_3_ profile. (**A**) Experimental design and longitudinal elevation in serum creatinine in CKD rats compared to control. Mean/SD. (**B**) 25(OH)D_3_, (**C**) 24,25(OH)_2_D_3_, (**D**) 25-VMR 24,25(OH)_2_D_3_:25(OH)D_3_, (**E**) 1,25(OH)_2_D_3_, (**F**) 1,24,25(OH)_3_D_3_, (**G**) 1,25-VMR 1,24,25(OH)_3_D_3_:1,25(OH)_2_D_3_. For (**B**–**G**) Repeated measures one-way ANOVA with post hoc test for differences between adjacent time points (0–2, 2–4, 4–5 weeks).

To evaluate the progression of metabolite changes longitudinally with the development of CKD, we measured the same vitamin D metabolite profile in rats with adenine-induced CKD and time-control healthy rats, as confirmed by elevations in serum creatinine (Fig. 3A). The longitudinal data demonstrated unchanged levels of 25(OH)D with progression of CKD (Fig. 3B) and a progressive decline in 24,25(OH)_2_D_3_ (Fig. 3C) and the 25-VMR (Fig. 3D). The level of 1,25(OH)_2_D_3_ and 1,24,25(OH)_3_D_3_ decreased progressively with declining kidney function (Fig. 3E,F). However, aligned with our observation in humans, the ratio between 1,24,25(OH)_3_D_3_ and 1,25(OH)_2_D_3_ increased with the induction and progression of CKD (Fig. 3G). As expected, blood levels of phosphate, PTH and FGF-23 increased as CKD progressed (Supplementary Fig. 1).

## Discussion

This study reports 1,24,25(OH)_3_D_3_ levels in humans across a spectrum of measured GFR. To address the limitations of a cross-sectional study, we evaluated the same parameters longitudinally in the circulation of rats with the induction and progression of CKD. 1,25(OH)_2_D_3_ catabolism reflected by the 1,25-VMR increased whereas 25(OH)D_3_ catabolism (25-VMR) decreased as mGFR declined, a finding that we also observed longitudinally in rats.

The progressive reduction in the 25-VMR with decreasing mGFR in humans, and with the induction and progression of CKD in rats, supports the work of others^12^ and indicates stagnation of 25(OH)D_3_ catabolism_._. The biological significance of 24-hydroxylated metabolites in the circulation is unclear; however, there is evidence that 1,24,25(OH)_3_D_3_ is biologically active and 24,25(OH)_2_D_3_ facilitates fracture repair^13–16^. Global reduction of 24-hydroxylation in CKD has been proposed to explain low levels of 24,25(OH)_2_D_3_, however, this theory requires careful further evaluation given that the 1,25-VMR does not trend similarly to the 25-VMR. The kidneys are the main site of origin of 1,25(OH)_2_D_3_ in the circulation, yet increased or unchanged expression of CYP24A1 in kidney tissue has been reported^17–20^. While those finding are not consistent with the reduction in the 25-VMR, they are consistent with the 1,25-VMR.

Participants taking cholecalciferol had higher levels of 1,24,25(OH)_3_D_3_. One possibility is that cholecalciferol may have increased 1,25(OH)_2_D_3_ production, and subsequent catabolism, despite low kidney function. If this hypothesis were true, this could support the use of cholecalciferol in patients with kidney disease. Alternatively, it could also suggest the presence of pathways that convert 25(OH)D_3_ to 1,24,25(OH)_3_D_3_ independently of a 1,25(OH)_2_D_3_ intermediary as suggested by Martineau et al.^16^ In a study of participants with moderate to severe CKD, cholecalciferol supplementation increased levels of 25(OH)D_3_ substantially, but the change in 24,25(OH)_2_D_3_ was more than proportional to the increase in 25(OH)D suggesting that supplementation increased delivery of 25(OH)D to CYP24A1 and/or increased CYP24A1 activity^21^. Levels of 1,25(OH)_2_D_3_ did not change. In a study involving participants of the Multi-Ethnic Study of Atherosclerosis, lower 25(OH)D and a lower 25-VMR was associated with a greater treatment response to cholecalciferol supplementation, as assessed by a change in PTH^22^. Similar to the previous study, there was no change in 1,25(OH)_2_D_3_ in the cholecalciferol supplemented participants.

Study strengths include the LC–MS/MS assessment of vitamin D metabolites which allows for accurate assessment of these structurally very similar metabolites, a limitation of other methods^4,23^ as well as the measurement of GFR, as opposed to estimation based on endogenous markers. The cross-sectional design of the human study limits interpretation, however the longitudinal rat data demonstrated the identical evolution of changes in vitamin D metabolites with progressing CKD. The participants were predominantly white limiting generalizability. Future studies should consider acute challenges of different metabolites to assess specificity and kinetics of transformation. We acknowledge that variability in metabolite-to-parent ratios may not solely reflect true enzymatic activity. For example, Hsu et al. demonstrated higher 25(OH)D clearance in Black individuals, despite lower a 25-VMR^24^. Further, given 1α-hydroxylated metabolites circulate at ~ 1000× lower concentration than their non-1α-hydroxylated counterparts, differences unrelated to 24-hydroxylation, for example intra-individual variation or assay variability, may meaningfully alter the 1,25-VMR, without altering other metabolites circulating at a higher-levels, and thus the circadian rhythm and the metabolism of 1,24,25(OH)_3_D_3_ is an important area of future research before use as a potential diagnostic tool. The development of a vitamin D profile that includes VMRs and evaluates the response over time to supplementation may better define adequacy in this population and guide future therapy as suggested by Melamed et al.^25^.

In summary, the circulating 24-hydroxylated 1,25(OH)_2_D_3_ ratio does not decrease like the 25-VMR as kidney function declines. The clinical implications of the divergence between catabolism of 25(OH)D_3_ versus 1,25(OH)_2_D_3_ requires further study. Understanding vitamin D catabolism and the potential that 24-hydroxylation products may have biological activity may help inform treatment strategies in the future.

## Supplementary Information

Supplementary Information.

## Data Availability

Data lies with the Principal Investigators and can be made available upon request.

## Abbreviations

1,25-VMR: 1,24,25(OH)_3_D_3_-to-1,25(OH)_2_D_3_ vitamin D metabolite ratio
25-VMR: 24,25(OH)_2_D_3_-to-25(OH)D_3_ vitamin D metabolite ratio
