# Supplementary material for: The metabolism of 1,25(OH)2D3 in clinical and experimental kidney disease
Source: Sci Rep. 2022 Jun 28;12:10925. doi: 10.1038/s41598-022-15033-9 (PMC9240002; doi:10.1038/s41598-022-15033-9)
Supplement: Supplementary file 1 — Supplementary Information. [file 41598_2022_15033_MOESM1_ESM.pdf]

Supplementary Information File

The metabolism of 1,25$(OH)_2D_3$ in clinical and experimental kidney disease

Mandy E Turner PhD[1], Tyler S Rowsell BSc[1], Christine A White MD[2], Martin Kaufmann PhD[1], Patrick A Norman MSc[3,4], Kathryn Neville BSc[1], Martin Petkovich PhD[1], Glenville Jones PhD[1], Michael A Adams PhD[1,2], Rachel M Holden MD*[1,2]

[1]Department of Biomedical and Molecular Sciences, Queen's University, Kingston, ON, K7L 3V6, Canada

[2]Department of Medicine, Queen's University, Kingston, ON, K7L 3V6, Canada

[3]Kingston General Health Research Institute, Kingston Health Sciences Centre, Kingston, ON, K7L 3V6, Canada

[4]Department of Public Health Sciences, Queen's University, Kington, ON, K7L 3V6, Canada

Supplementary Table S1: Spearman correlations of vitamin D metabolites non-dialysis controlling for age and mGFR unadjusted and controlling for age and mGFR

Variable	$25D_3$	$24,25D_3$	$1,25D_3$	$1,24,25D_3$	$24,25D_3$ to $25D_3$	$1,24,25D_3$ to $1,25D_3$
Unadjusted correlations						
$25D_3$	-	0.67	0.26	0.33	0.24	-0.01
$24,25D_3$	0.67	-	0.43	0.56	0.86	-0.03
$1,25D_3$	0.26	0.43	-	0.35	0.39	-0.65
$1,24,25D_3$	0.33	0.56	0.35	-	0.51	0.40
$24,25D_3$: $25D_3$	0.24	0.86	0.39	0.51	-	-0.03
1,24,25D: $1,25D_3$	-0.01	-0.03	-0.65	0.40	-0.03	-
$1,25D_3$ to $25D_3$	-0.33	0.02	0.78	0.11	0.25	-0.64
$1,25D_3$ to $24,25D_3$	-0.46	-0.57	0.38	-0.25	-0.46	-0.54
Partial correlations controlling for age and mGFR						
$25D_3$	-	0.75	0.35	0.32	0.27	-0.05
$24,25D_3$	0.75	-	0.19	0.46	0.80	0.14
$1,25D_3$	0.35	0.19	-	0.21	-0.05	-0.58
$1,24,25D_3$	0.32	0.46	0.21	-	0.40	0.57
$24,25D_3$: $25D_3$	0.27	0.80	-0.05	0.40	-	0.25
1,24,25D: $1,25D_3$	-0.05	0.14	-0.58	0.57	0.25	-

Spearman r value. Colour delineates p-value: Red<0.001, Yellow <0.01, Green<0.05

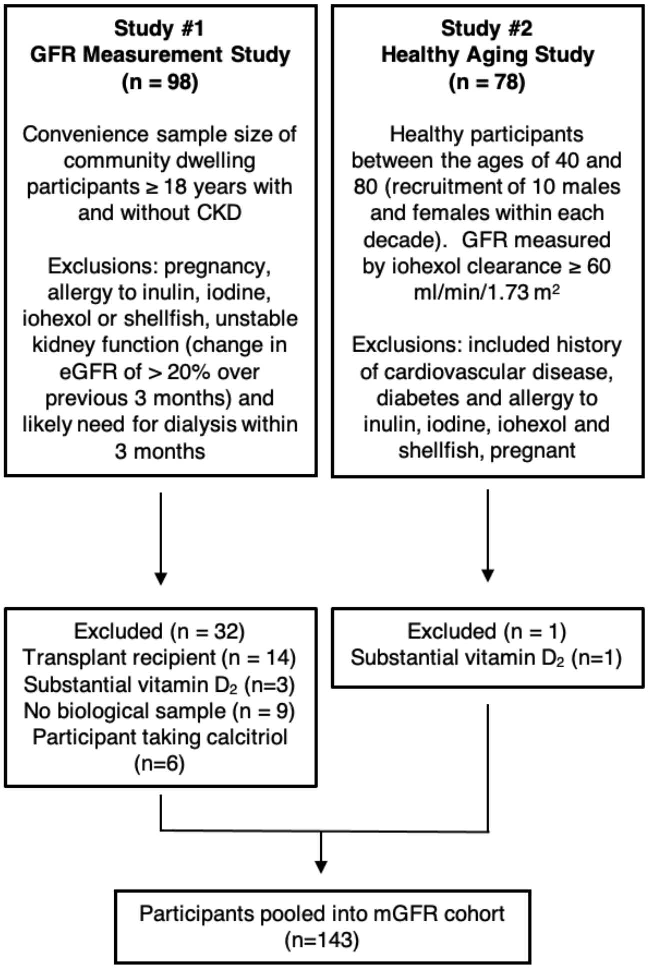

Supplementary Figure S1: Description of the two studies from which the present cohort was derived.

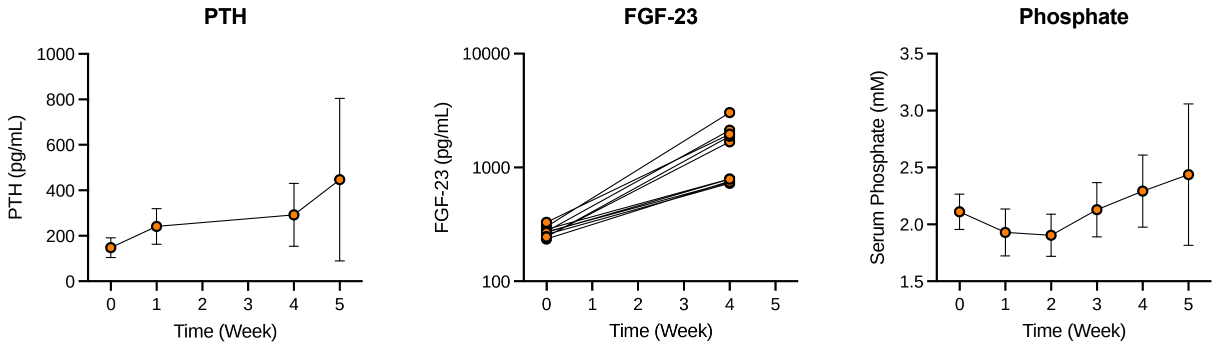

Supplementary Figure S2: Longitudinal CKD serum phosphate, parathyroid hormone (PTH), and fibroblast growth factor 23 (FGF-23) in a rat model of CKD.
